# Maternal and congenital toxoplasmosis, currently available and novel therapies in horizon

**DOI:** 10.3389/fmicb.2014.00385

**Published:** 2014-07-24

**Authors:** Helieh S. Oz

**Affiliations:** Department of Medicine, University of Kentucky Medical CenterLexington, KY, USA

**Keywords:** fetal maternal, congenital toxoplasmosis, mind alteration, sexual transmission, atovaquone, diclazuril

## Abstract

Over one billion people worldwide are predicted to harbor *Toxoplasma* infection frequently with unknown lifelong health consequences. Toxoplasmosis is an important cause of foodborne, inflammatory illnesses, as well as congenital abnormalities. Ubiquitous *Toxoplasma* has a unique tropism for central nervous system with a mind-bugging effect and is transmitted sexually through semen. Currently available therapies are ineffective for persistent chronic disease and congenital toxoplasmosis or have severe side effects which may result in life-threatening complications. There is an urgent need for safe and effective therapies to eliminate or treat this cosmopolitan infectious and inflammatory disease. This investigation discusses pathogenesis of maternal and congenital toxoplasmosis, the currently available therapies in practice, and the experimental therapeutic modalities for promising future trials.

## INTRODUCTION

**FIGURE 1:**
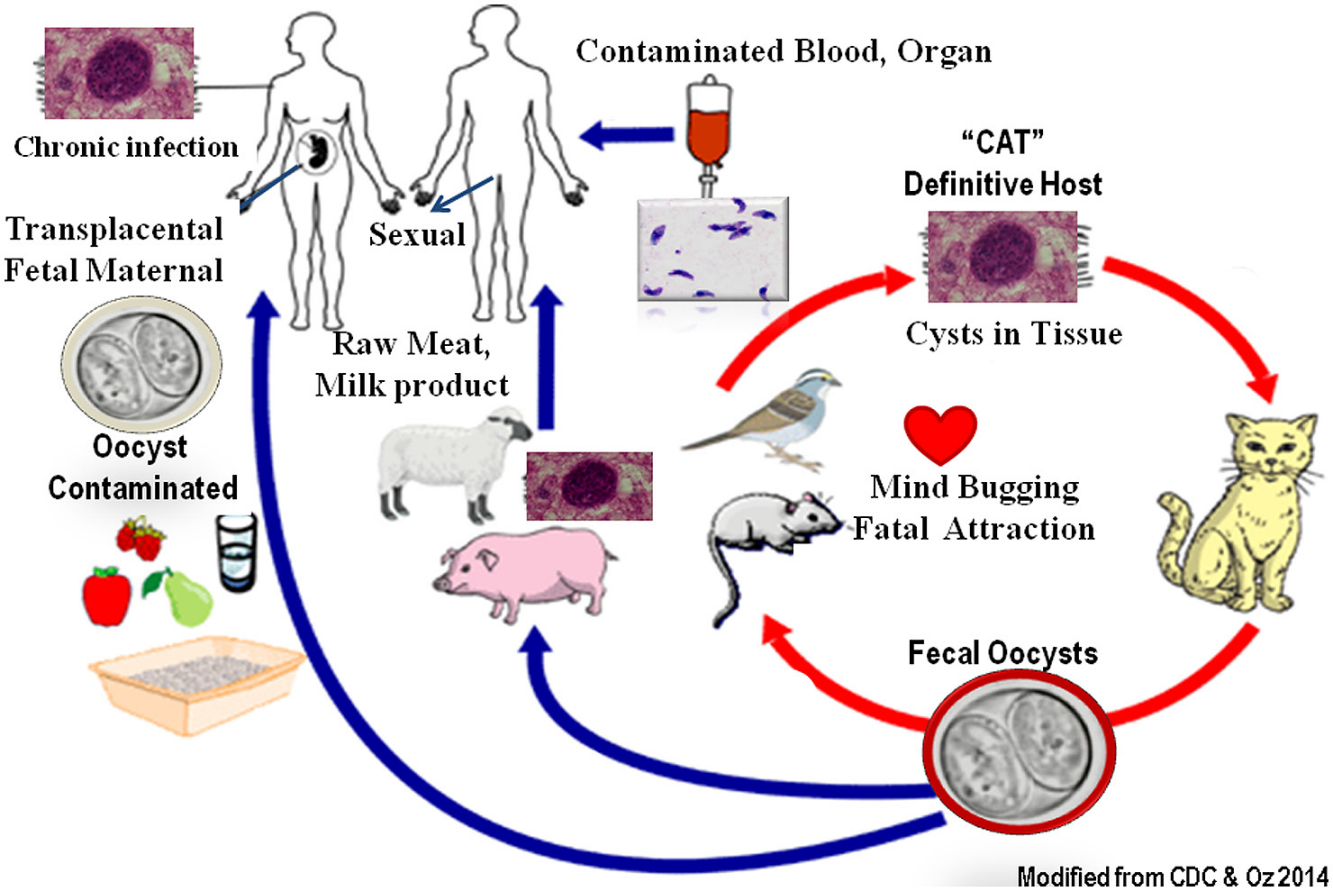
**Multiple sources of ubiquitous *Toxoplasma* for maternal congenital transmission**.

Over one billion people worldwide are predicted to harbor *Toxoplasma* infection frequently with unknown lifelong health consequences. Toxoplasmosis is one of the most important foodborne inflammatory illnesses, as well as congenital abnormalities (Hoffmann et al., 2012). *Toxoplasma* is classified as “Category B pathogen” which once infected, the organisms dwell in organs such as muscles and brain in cyst forms for the life of the patient/host to become reactivated. The organisms have a sexual stage in cat’s intestinal epithelial cells which form resistant oocysts passed in feces and matured in dirt (**Figure 1**). Humans and other animals develop systemic infection in asexual form by ingestion of contaminated vegetable, fruits, water, or consumption of infected milk and undercooked sea food, poultry, and livestock. Tachyzoites infect nucleated host cells and utilize monocytes, macrophages, and dendritic cells as “Trojan Horse” (1) to escape the host immune defense (Elsheikha and Khan, 2010), (2) to bypass the blood–brain barrier (Bierly et al., 2008) and the placenta barricade, and (3) to spread and form systemic disease. *Toxoplasma* infests particularly rural and impoverish communities of women, African American, Hispanics, and Native Americans as a “frequently ignored disease of poverty” (Hotez, 2008). Toxoplasmosis is considered as the second major cause of foodborne death in the United States (Scallan et al., 2011). The *Toxoplasma* annual cost of illnesses is about $3 billion and the quality-adjusted life loss is equal to 11,000 years in the United States (Hoffmann et al., 2012). Toxoplasmosis in immune-intact individuals is generally symptomless and undetected or appears like flu syndrome and malaise. However, it can cause severe pathological consequences in immunocompromised patients, fetuses, and neonates and lead to demise and death (Dubey and Jones, 2008).

## MATERNAL CONGENITAL TOXOPLASMOSIS

The importance of maternal and congenital transmission has long been recognized since 1939; when a neonate from New York developed toxoplasmosis (Wolf et al., 1939; Jones et al., 2001). During progression of pregnancy, maternal immune system confronts a dual predicament: the growing embryo, and the environmental toxins and pathogens threatening mom and fetus. In fact, successful pregnancy involves an elegant equilibrium in organizing the immune system at the fetal-maternal and uteri milieu resulting in tolerance (TH2) of the fetus (Norwitz et al., 2001; Muzzio et al., 2014) and defense (TH1) against the pathogenic agents. Women who have acute or reactivated toxoplasmosis during pregnancy can transplacentally transmit organisms to their fetus. As, tachyzoites bypass the placental blood barrier and invade the fetal organs to propagate and compromise the embryonic developmental process. About 50–80% of child-bearing Brazilian women and 50% of children have anti-*Toxoplasma* antibodies. Also, 5–23 neonates are found to be infected per 10,000 in Brazil (Dubey et al., 2012).

Congenital toxoplasmosis can manifest with severe complications, such as miscarriage, fetal developmental retardation, encephalitis, neurological, mental illnesses, visual, and auditory inflammatory disorders, cardiovascular abnormalities, and pains (Dunn et al., 1999; Gilbert and Gras, 2003; Gras et al., 2005; McLeod et al., 2006; Remington et al., 2006; Oz and Tobin, 2012). The severity of complications relies on the gestation period, as the early infection shows more severe outcomes (Dunn et al., 1999; Remington et al., 2006). While, fetuses infected in late gestation are born normal, may develop central nervous system (CNS) symptoms and retinochoroiditis later in life. Also, the new lesions may occur in untreated as well as treated children (Dunn et al., 1999).

A predominant source of infection in North America is contaminated food and water with oocysts passed in the cat’s (definite host) feces (Boyer et al., 2011). Sera and surveys from 76 moms with congenital infected newborns were collected from four different epidemic areas and investigated by the National collaborative Chicago-based congenital toxoplasmosis. The data revealed 78% of the moms acquired primary infection from oocysts form, while only 49% had direct contact with house cats. Hence, extensive educational hygienic programs, effective cats’ infection prevention, and vaccination plans, along with serological testing of pregnant women and newborns, followed by the treatments are needed to prevent maternal congenital toxoplasmosis (Boyer et al., 2011).

## MATERNAL REACTIVATION AND CONGENITAL TOXOPLASMOSIS

Toxoplasmosis reactivation is a major concern in pregnant, immunodeficient, blood transfusion, bone marrow, and organ transplant patients, when the protective cyst’s wall ruptures and organisms reach the lymphatic and blood cells to activate and propagate the infection. Maternal congenital toxoplasmosis is instigated by the transplacental transmission of organisms in maternal infection (Buxton et al., 1991), as *Toxoplasma* organisms alter balance in immune milieu leading to inflammatory response. A low number of *Toxoplasma* organisms can induce an extensive inflammatory and immune reaction as shown in the murine model of fetomaternal toxoplasmosis (Oz and Tobin, 2012). Therefore, taming exaggerated inflammatory response in fetal–maternal toxoplasmosis is necessary to prevent severe tissue destruction and fatality during the pathogenic clearance.

According to Massachusetts Department of Health, about 1 case of congenital toxoplasmosis occurs for every 10,000 live births. It is estimated that from 4,000,000 live births each year in the United States, 400 have acquired congenital toxoplasmosis (Mead et al., 1999). This rate is extensively higher for other developing countries. For instance, retrospective trials from Argentina (2000–2011) reviled 18% (2206/12035) prevalence rate of anti-*Toxoplasma* antibody in pregnant women. Thirty eight per 10,000 of these moms had developed acute infection and 5.8% transplacentally infected their neonates (Carral et al., 2013).

## TOXOPLASMA MIND-BUGGING SEXUAL ATTRACTION AND MENTAL DISORDERS

Recent investigations reveal that *Toxoplasma* provokes a brain and mind alteration with sexual arousal in rats seeking cat, while uninfected normal rats fear and avoid predator’s urine odor with an immediate, innate survival defensive behavior (House et al., 2011; Knight, 2013). Therefore, the brain impaired and fearless infected rodents are eaten up by feline to fulfill the organism’s sexual propagation in definitive host “cat”. *Toxoplasma* manipulates the limbic brain neurons responsible for instinct defensive response and augments activity in adjacent limbic regions of sexual desire when exposed to cat’s urine odor (House et al., 2011).

Toxoplasmosis is a sexually communicable disease as organisms are transmitted by contaminated semen during natural mating. Also, artificial insemination with contaminated semen can infect animals with vertical transmission with 80% embryonic disruption (Arantes et al., 2009; Lopes et al., 2013; Wanderley et al., 2013). Indeed, there exists a potential sexual transmission route with infected semen during mating as well as artificial insemination with subsequent vertical transmission to the progeny in humans.

*Toxoplasma* has strong tropism for the CNS with adverse affect in the brain neuro-structural development and pathological as well as psycho-behavioral impairment and mental challenges (Bachmann et al., 2005; Brown et al., 2005; Wang et al., 2006). Maternal *Toxoplasma* infection has been related with risk for schizophrenic events and autism with over 40 supporting investigations for the incidence of *Toxoplasma* infection among these patients (Flegr, 2013). *Toxoplasma* infection may evolve brain dopamine dysregulation (Torrey et al., 2007; Fekadu et al., 2010). Longitudinal and cross-sectional trials in seropositive females with chronic toxoplasmosis have shown high risk of self-harm, accidents and non-fatal suicidal aggression than in seronegative individuals (Pedersen et al., 2012; Zhang et al., 2012).

Pregnant women with latent infection have a higher risk of infants with genetic or developmental disorders such as premature and postnatal slow motor development due to infection provoked immunosuppression in moms. Some of these defects are related to malnutrition caused by diarrhea and gut disorders or directly congenital toxoplasmosis induced cognitive and developmental deficits (Kankova et al., 2012).

## AUTOIMMUNE DISEASE AND TOXOPLASMOSIS

Ubiquitous *Toxoplasma* infection is indicated to provoke series of chronic inflammatory and autoimmune disorders. Immunosuppressants and monoclonal antibodies such as anti-TNF which are widely used in healthcare for the treatment of autoimmune diseases, and organ transplantation may result in acute toxoplasmosis in these patients. However, the nature of this interaction and mechanism between the development of acute toxoplasmosis and immunosuppressant therapies are still being investigated. In a clinical trial, sera of 1514 patients with 11 different autoimmune diseases from health centers in Europe and Latin America and 437 matched controls examined for the prevalence of anti-*Toxoplasma* antibodies, IgG and IgM and auto-antibodies (Shapira et al., 2012). Fourty-two percent of patients had anti-*Toxoplasma* antibody IgG, versus 29% of those without autoimmune complications (*p* < 0.0001). Anti-*Toxoplasma* antibody IgG was associated with anti-phospholipid syndrome, autoimmune thyroid diseases, systemic sclerosis, and rheumatoid arthritis (*p* < 0.0001). Anti-*Toxoplasma* antibody IgM was more prevalent in patients with anti-phospholipid syndrome (*p* < 0.01), systemic sclerosis (*p* < 0.05) and inflammatory bowel disease (IBD; *p* < 0.05) than in controls. These findings strongly support that *Toxoplasma* may contribute to the autoimmune disease pathogenesis (Shapira et al., 2012).

Crohn’s disease and ulcerative colitis (IBD) are considered as autoimmune response of a leaky gut to microbiota, when toxins, like Gram-negative lipopolysaccharide (LPS), bypassing the inflamed epithelia by underlying dysregulated oral tolerance (Brandtzaeg, 2001; Oz et al., 2009, 2013). The use of biological blockers, like anti-TNF antibodies, and immunosuppressives in IBD patients increases the risk of opportunistic diseases (Oz and Ebersole, 2009). Crohn’s patients are prone to intestinal abscess formation including *Toxoplasma* infection (Epple, 2009). Dams infected with *Toxoplasma* develop severe colonic inflammatory response with significant shortening in colonic length, infiltration of lymphocytes, and macrophages and microabscess formations in the cryptic microstructures resembling Crohn’s pathogenesis (Oz and Tobin, 2012, 2014; Oz, 2014). Similar to colitis, *Toxoplasma*-induced ileitis elevates precarious gut microbial, LPS and lipopeptide contents (Erridge et al., 2010). Anti-*Toxoplasma* titer has been detected significantly higher in IBD patients than healthy controls; supporting the notion that toxoplasmosis to trigger IBD and specifically Crohn’s disease pathogenesis in patients (Lidar et al., 2009). *Toxoplasma* infection causes an excessive Th1 systemic inflammation and promotes pro-atherogenesis (Portugal et al., 2009; Lige et al., 2013). Therefore, inflammatory response may act as a dual-edged sword. While, necessary for the host defense and recovery against pathogens, unleashed exaggerated chronic inflammation against *Toxoplasma* infection causes loss of function in organs as seen in the autoimmune syndrome (Carter, 2013).

## DIAGNOSIS OF MATERNAL CONGENITAL TOXOPLASMOSIS

Maternal *Toxoplasma* infection as a serious risk factor for the fetus requires accurate and urgent diagnosis for possible prevention and treatments. Maternal congenital toxoplasmosis is commonly diagnosed with utilizing repeated serological tests to assess the types and the levels of anti-*Toxoplasma* antibodies. Pregnant moms are required to be tested in Austria, France, Italy, Portugal, and Uruguay for antibody detections, but a limited screening program is used in Belgium, Germany, and Switzerland. Congenital and neonatal screening for toxoplasmosis is performed in over two million women and their babies each year in Europe, North and South America with estimated cost of over 500 million dollars (Petersen and Schmidt, 2003). While, the United States does not require routine screening, it is recommended that infants with serious systemic complications to be tested for toxoplasmosis (Armstrong et al., 2004). In addition, seronegative pregnant women indicating no previous exposure to infection are at risk for the infection and recommended to be serology tested monthly until the labor.

Diagnosis of toxoplasmosis is based on the presence of IgM and IgG anti-*Toxoplasma* antibodies, and molecular techniques to detect organisms (Teixeira et al., 2013). Acute infection is associated with high levels of anti-*Toxoplasma* IgM antibody followed by a rise in IgG levels in 1–3 weeks. Detection of IgM or elevation of IgG anti-*Toxoplasma* antibodies suggests acute or reactivation with a possible transmission of infection to the fetus. An amniotic fluid test is required to confirm fetal health status and possible exposure to the maternal infection.

Sabin-Feldman dye test “the international gold standard” is a complement-lysis-based assay and relatively sensitive and specific for anti-*Toxoplasma* IgG antibody. The test is considered more reliable than available ELISA kits, but requires live organisms treated with each diluted serum analyzed under the microscope (Dando et al., 2001).

In infants with neurological disorders, anti-*Toxoplasma* IgM and IgA antibodies plus cerebrospinal fluid PCR to detect *Toxoplasma* DNA are considered to provide a high sensitivity for diagnosis of congenital toxoplasmosis (Olariu et al., 2014). CSF-PCR was positive in 47% of about 60 infants from infected moms, while 0% positive in uninfected healthy ones.

Additionally, western blot analysis is used to detect IgM and IgA (di Carlo et al., 2011) and RT-PCR for DNA in amniotic fluid with 98% sensitivity and 100% specificity (Teixeira et al., 2013).

### α-FETOPROTEIN SERUM ANALYSIS

α-Fetoprotein, released by embryonic hepatic cells, is a biomarker to predict development and birth defects, and useful in prediction of fetoplacental health and growth progression. Altered levels of maternal α-fetoprotein are associated with pregnancy, hepatic complications, tumors, fetal demise, and resorption (Mizejewski, 2007) and may be found useful in prediction of immune responses and intrauterine death in toxoplasmosis (Kaur and Verma, 1995; Oz, 2014).

## CURRENTLY AVAILABLE AND NOVEL ANTI-TOXOPLASMOSIS THERAPIES IN HORIZON

Toxoplasmosis is a forgotten disease with no safe and effective therapy available for chronic persistent or pernicious fetal–maternal infection. Spiramycin has been used in feto-maternal toxoplasmosis prevention and treatment in Canada, Latin America, and Europe for decades but is classified under “experimental therapy” in the United States. Spiramycin monotherapy is effective in early pregnancy as a preventive measure but not after fetal exposure to the infection. In a prospective cohort trial in Brazil, 58% of newborns from spiramycin-treated moms, in contrast to over 73% from untreated ones had congenital infection (Avelino et al., 2014). More than 50% of patients treated with spiramycin retained *Toxoplasma* DNA in peripheral blood and remained infected (Habib, 2008). In another clinical trial of the neonates from infected moms treated with spiramycin and pyrimethamine plus sulfadoxine in France, 24% of 257 children were diagnosed with congenital infection. Of these, 7% were predicted to be infected in the first, 24% in the second, and 59% in the third trimesters, respectively (Bessières et al., 2009). Other fetal–maternal treatments are azithromycin, clarithromycin, atovaquone, dapsone, and cotrimoxazole (trimethoprim–sulfamethoxazole), however, their efficacy has not been proven (Petersen and Schmidt, 2003).

## ATOVAQUONE AND MATERNAL CONGENITAL

Atovaquone, a hydroxy-1,4-naphthoquinone and FDA approved, is fairly safe and effective treatment against tachyzoites and cyst forms of *Toxoplasma* and anti-*Plasmodial* (Hudson et al., 1991; Dunay et al., 2004). It is used in adults, yet not approved for fetal–maternal and children toxoplasmosis (Cortina-Borja et al., 2010). Atovaquone is anti-fungal *Pneumocystic* pneumonia and anti-*Babesia microti,* causative of human blood-borne babesiosis endemic in New England and North Eastern in the United States (Hughes and Oz, 1995; Oz and Westlund, 2012). Atovaquone acts by targeting mitochondrial respiration and binds to the ubiquinol oxidation on cytochrome bc1 complex to block and to collapse the membrane in the organisms (Srivastava and Vaidya, 1999; Freyre et al., 2008). Atovaquone has a half-life of 1.5–3 days and mainly binds to plasma proteins (99%) and is excreted into feces (94%) without being metabolized (Rolan et al., 1997). Atovaquone has been shown to protect against maternal congenital toxoplasmosis and inflammatory complications in murine model (Oz and Tobin, 2012). Atovaquone was superior than the standard of care with combined pyrimethamine plus sulfadiazine or pyrimethamine plus clindamycin therapies against brain inflammatory responses and the severity of infection in the mice (Dunay et al., 2004).

## MATERNAL CONGENITAL TOXOPLASMOSIS AND DICLAZURIL

Diclazuril [4-chlorophenyl [2,6-dichloro-4-(4,5-dihydro-3*H*-3,5-dioxo-1,2,4-triazin-2-yl)phenyl acetonitrile] is related to herbicides and used to protect poultry and livestock against coccidiosis, induced gastroenteritis, morbidity, and mortality. *Toxoplasma and* coccidians are members of the phylogeny *Apicomplexan* with a highly conserved region of protochlorophyllide with traces of plant chloroplast epitope not present in humans and animals. Apicoplast is an extranuclear DNA organelle containing transcriptional and translational device in *Toxoplasma* with specific enzymes unwinding DNA. It is presumably originated from eukaryotic *Ciliate* ancestors and prokaryotic green alga in evolution (Köhler et al., 1997). Apicoplast has a unique sensitivity to herbicidal agents with a safe and attractive region for drug discovery and vaccine target in *Toxoplasma* metabolic pathway, absent in the humans and animals.

Diclazuril and its related compounds specifically invade and attach to chloroplast epitope and the D1 protein of the *Toxoplasma* apicoplast without interacting to damage the mammalian host organs (Hackstein et al., 1995). Additionally, diclazuril can downregulate expression of serine/threonine protein phosphatase in merozoites of *Eimeria* to induce apoptosis with possible mechanism of action against *Toxoplasma* organisms (Zhou et al., 2013; Oz, 2014).

Diclazuril is a non-toxic agent (Assis et al., 2010) with rapid absorption following oral administration to reach a constant level in plasma and cerebrospinal fluid. Recent studies have shown diclazuril to be well tolerated and effective in murine model for maternal and congenital toxoplasmosis (Oz and Tobin, 2014). While atovaquone protects against some aspects of gastrointestinal complications in experimental congenital toxoplasmosis in murine (Oz and Tobin, 2012), diclazuril was superior than atovaquone in improving anemia, colonic length, and hepatic complications against maternal toxoplasmosis (Oz and Tobin, 2014). In addition, diclazuril and atovaquone combination therapy is anticipated to exert a unique synergistic effect against toxoplasmosis. Diclazuril monotherapy or combination with atovaquone therapy may warrant clinical trials in maternal congenital as well as in ocular and chronic toxoplasmosis. Finally, diclazuril is anticipated to be used as a novel protective and preventive measure to eliminate the cycle of *Toxoplasma* infection in the definitive host, feline.

## Conflict of Interest Statement

The author declares that the research was conducted in the absence of any commercial or financial relationships that could be construed as a potential conflict of interest.
